# Methodology for pediatric head computed tomography image segmentation and volumetric calculation using a tablet computer and stylus pen

**DOI:** 10.1007/s00381-024-06723-y

**Published:** 2024-12-23

**Authors:** H. Hashimoto, M. Shimada, O. Takemoto, Y. Chiba

**Affiliations:** 1 Department of Neurosurgery, Osaka Women’s and Children’s Hospital, Izumi, Osaka 594–1101 Japan; 2 Department of Neurological Diagnosis and Restoration, Graduate School of Medicine, Osaka University, Suita, Osaka 565–0871 Japan; 3 Center for Neurotechnology and Neurorecovery, Department of Neurology, Massachusetts General Hospital, Boston, MA 02114 USA; 4 Department of Neurology, Harvard Medical School, Boston, MA 02115 USA; 5 Department of Radiology, Osaka Women’s and Children’s Hospital, Izumi, Osaka 594–1101 Japan

**Keywords:** Image segmentation, Volumetric measurement, Tablet computer, Stylus pen

## Abstract

**Purpose:**

This study presents a MATrix LABoratory (MATLAB)-based methodology for calculating intracranial volumes from head computed tomography (CT) data and compares it with established methods.

**Methods:**

Regions of interest (ROI) were manually segmented on CT images using a stylus pen, facilitated by mirroring a computer desktop onto a tablet. The volumetric process involved three main steps: (1) calculating the volume of a single voxel, (2) counting the total number of voxels within the segmented ROI, and (3) multiplying this voxel count by the single-voxel volume. This method was applied to 83 pediatric head CT scans from patients with minor head trauma, and the volumetric results were compared with those obtained from OsiriX.

**Results:**

A paired t-test revealed a statistically significant difference (*p* < 0.001) between volumes obtained with our MATLAB-based method and those from OsiriX, with our method measuring 0.32% higher. However, an unpaired t-test found no statistically significant differences between the volumetric population groups (*p* = 0.84).

**Conclusion:**

The significant difference identified by the paired t-test likely reflects statistical distinctions arising from differences in the calculation methods of the two approaches. Conversely, the unpaired t-test suggests no statistically detectable differences between the volumetric populations. Although this does not imply that the two methods produce identical results, the volumetric populations derived from our method may originate from the same underlying population as those obtained using OsiriX. By taking these points into account, our method has the potential to serve as a valuable tool for volumetric measurements.

**Supplementary Information:**

The online version contains supplementary material available at 10.1007/s00381-024-06723-y.

## Introduction

Volumetric measurements derived from computed tomography (CT) imaging [1] and magnetic resonance imaging (MRI) [2–4] have been widely utilized for quantitative research, including the development of normal growth curves for healthy children [2, 5, 6]. Additionally, volumetric assessments have been used to quantitatively evaluate patients with various conditions, such as Chiari malformation type I [3, 7] or type II [8, 9].

In the 1990s, intracranial volumetric measurements were roughly estimated by multiplying the transverse and anteroposterior diameters with the slice thickness obtained from CT images [1]. Subsequently, a system based on the C programming language was introduced, enabling segmentation of regions of interest (ROI) [10, 11]. The Cavalieri method has been applied to calculate the volumes of intracranial structures [12, 13], along with semi-automated segmentation programs such as the open-source software ITK-SNAP (the University of Pennsylvania, Philadelphia, PA, USA) [4]. Commercial software such as Amira (Thermo Fisher Scientific Inc., Waltham, MA, USA) [14], the Philips iSite enterprise System (Philips, Amsterdam, the Netherlands) [8], OsiriX (Pixmeo, Bernex, Switzerland) [15], and iPlan (Brainlab, Munich, Germany) [16, 17] have been employed for obtaining volumetric data. A custom tool developed using MATrix LABoratory (MATLAB, MathWorks, Natick, MA, USA) for calculating PCFV or ventricular volume has also been reported [2, 7].

The continuous advancement in volumetric measurement techniques has expanded the range of available tools, offering significant benefits but also presenting challenges for beginners in selecting the most suitable method. This study presents a MATLAB-based approach for segmentation and volumetric calculation of Digital Imaging and Communications in Medicine (DICOM) CT data using a tablet computer and stylus pen. Our results were compared with those obtained from other established tools, such as OsiriX. The MATLAB code is provided (see Supplemental Data), and we believe that this ready-to-use tool will be particularly advantageous for beginners seeking an accessible solution for volumetric analysis.

## Methods

### Patients and study setting

To evaluate our methodology, we calculated intracranial volume from head CT scans. We retrospectively enrolled DICOM-CT images from pediatric patients who underwent CT examinations for minor head trauma between March 2006 and May 2023 at Osaka Women’s and Children’s Hospital.

To minimize the influence of disease or trauma, we applied the following exclusion criteria: (1) suspicion of abuse, (2) need for craniotomy for decompression within a few days after a head injury, (3) cranial depressed fracture requiring surgery, (4) apparent compression or deformation of lateral ventricles due to hematoma, (5) Ommaya reservoir implantation or drainage surgeries, (6) presence of complications such as craniosynostosis, tumor, epilepsy, autism, intracranial arachnoid cyst, chromosomal abnormalities, cardiovascular disease, and endocrine disorder etc., and (7) presence of cavum Vergae or cavum septum. Therefore, all subjects were healthy before the head trauma and did not require any surgical intervention. Additionally, since pediatric intracranial volume increases rapidly and can grow to two to three times its size by the age of two years [6], we limited the inclusion criteria to subjects between two and ten years of age to minimize variability due to growth effects. The study population was derived from our previous research [5, 6].

### CT protocols

All CT scans were acquired with a 0.5 mm slice width, and images reconstructed with a 5 mm slice thickness were used for volume calculations. CT scans were performed using either Aquilion ONE or Aquilion Prime SP (Canon Medical Systems, Otawara, Japan). Due to the retrospective nature of this study, achieving consistency in CT protocols was not possible. The details of the CT protocols are presented in Supplemental Table 1.

### Volume calculation using our methodology

Our method for calculating the volume based on DICOM images is as follows: Firstly, we calculate the area of one pixel by multiplying its length and width, as recorded in the DICOM data. Next, we calculate the volume of one voxel by multiplying this area by the slice thickness (a pixel in three-dimensional space is called a voxel). Finally, we count the total number of pixels within the segmented ROI and calculate the volume of the segmented ROI by multiplying this total by the volume of one voxel. A similar methodology has been described in a previous study [11].

**Fig. 1:**
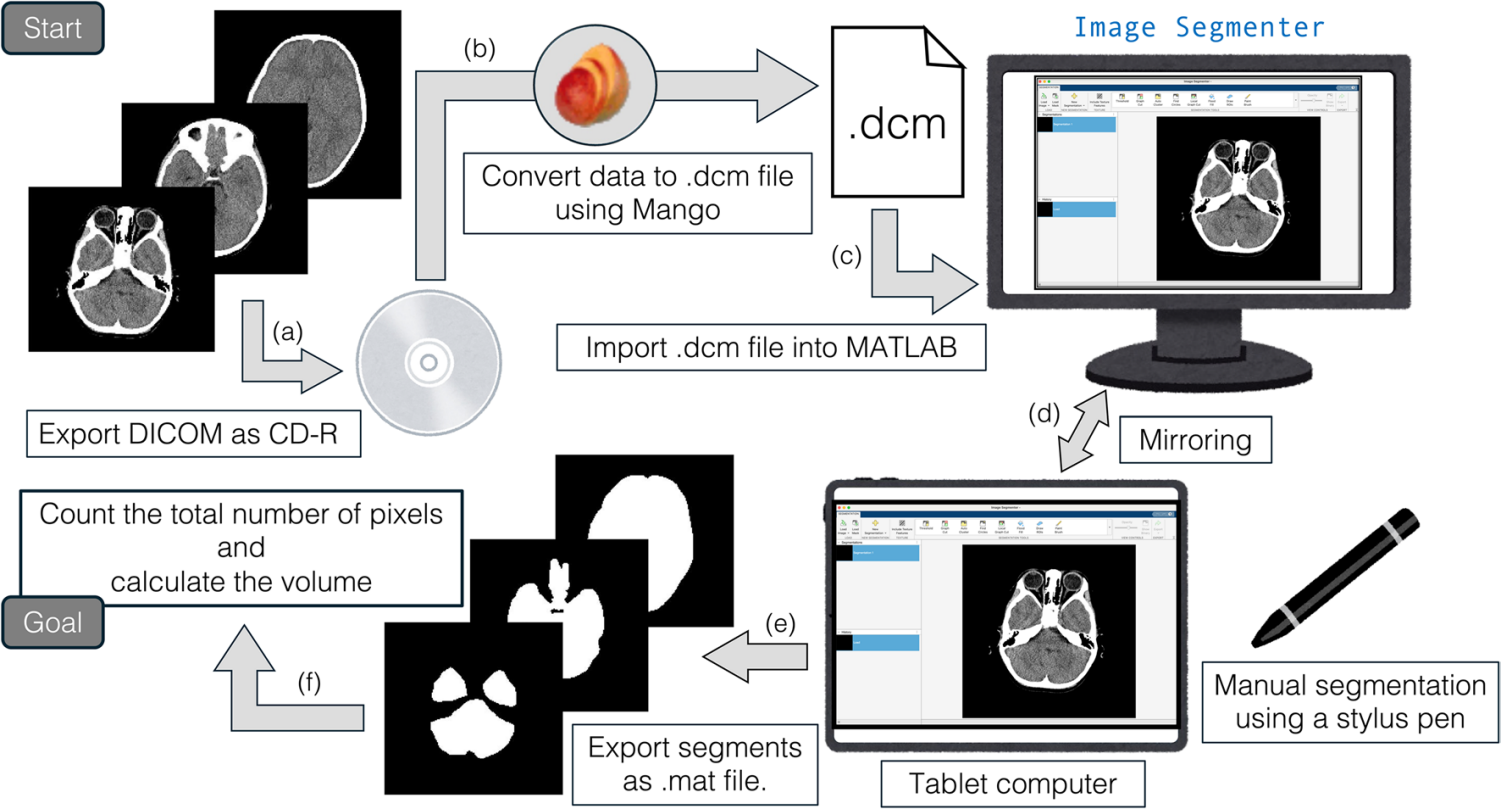
Schema for volume calculation. The DICOM data are initially exported via CD-R (**a**) and then converted into a.dcm file using Mango software (**b**), which can be imported into MATLAB (**c**). The Image Segmenter app is used to obtain ROI, and the desktop is mirrored to a tablet computer. The ROI is manually traced on the screen using a stylus pen (**d**). Once all ROI segments are obtained (**e**), their volume is calculated (**f**)

Details of our process for volume calculations are illustrated in Fig. 1. The DICOM data were initially exported to a compact disc-recordable (CD-R) at our hospital for security reasons (Fig. 1a). The DICOM data on the CD-R were loaded and exported as a single file in the.dcm format using Mango software (Multi-image Analysis graphical user interface, v4.1, available at https://mangoviewer.com/index.html) (Fig. 1b). The DICOM data in the.dcm format were then imported into MATLAB R2023a (Fig. 1c) using the Image Processing Toolbox. We utilized the Image Segmenter app in MATLAB (https://www.mathworks.com/help/images/ref/imagesegmenter-app.html). To manually segment the ROI from the original DICOM images, we used the imageSegmenter function implemented in the Image Processing Toolbox (https://mathworks.com/help/images/image-segmentation-using-the-image-segmenter-app.html). To facilitate this segmentation process and improve efficiency, we mirrored the desktop displaying the Image Segmenter app onto a tablet computer (in our case, a 12.9-inch iPad Pro 5th generation, Apple, Inc., Cupertino, CA). To obtain segmentation, we manually traced the ROI on the tablet computer screen using a stylus pen (in our case, Apple Pencil 2nd generation, Apple, Inc., Cupertino, CA) (Fig. 1d). Once all ROI segments were obtained (Fig. 1e), we counted the total number of pixels in the segments and calculated volumetric values (in milliliter, mL) (Fig. 1f). The segmentation process was performed by a single examiner (Hiroaki Hashimoto). MATLAB code related to this procedure is provided in the Supplemental Data.

**Fig. 2:**
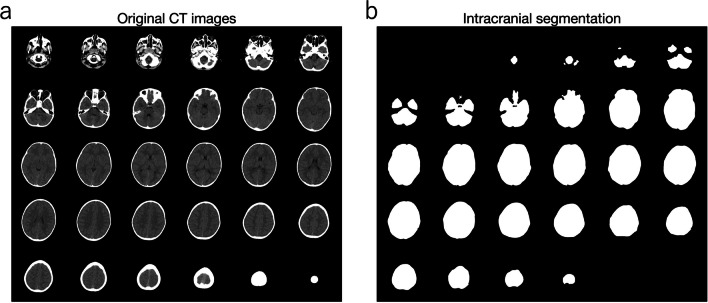
Representative segmentation. Head CT images acquired from a four-year-old male are presented (**a**), and segmentations focusing on the intracranial region are shown (**b**)

**Fig. 3:**
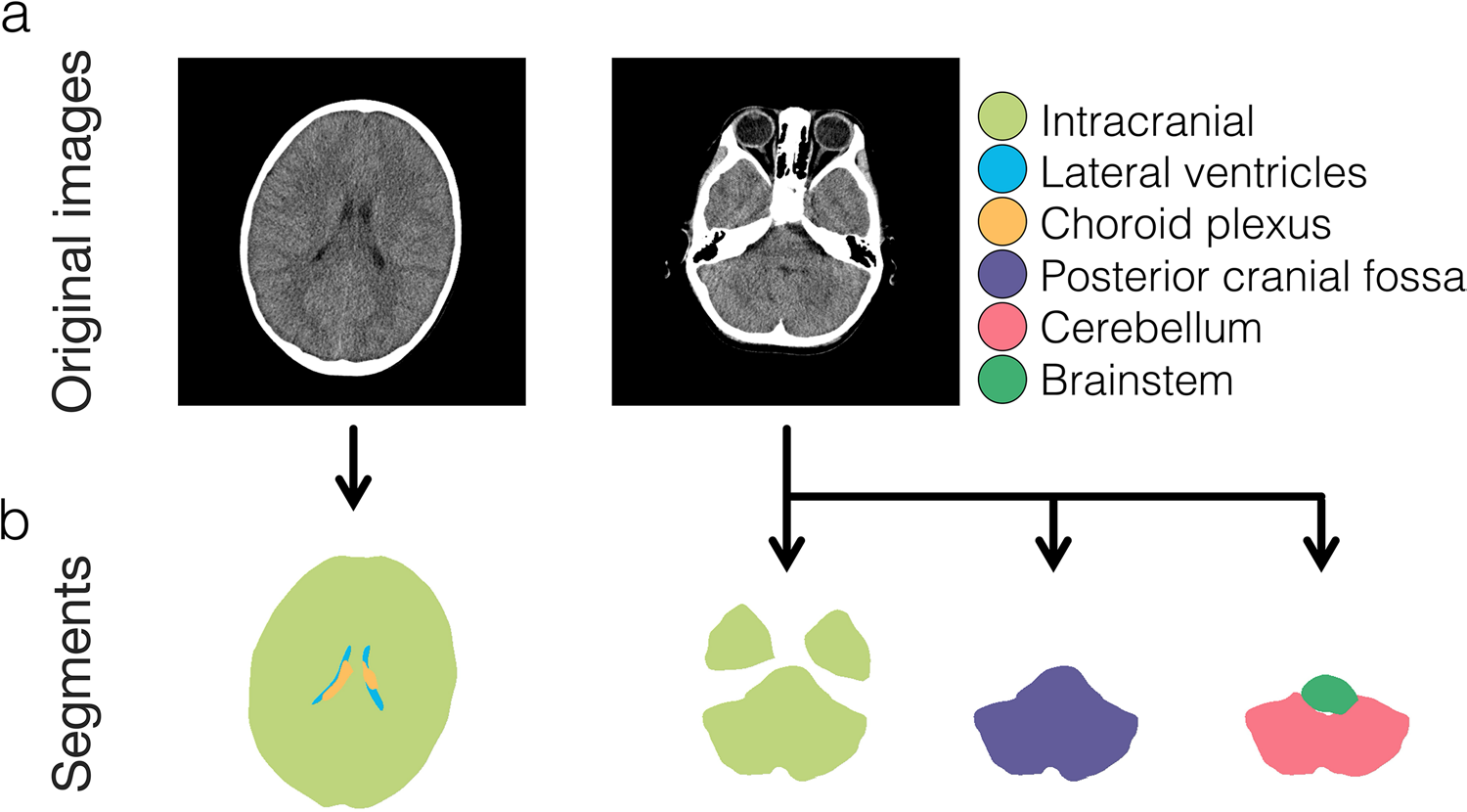
Segmentations other than the intracranial volume. The original DICOM images are displayed in the upper row (**a**). These images are the same as those shown in Fig. 2a. Representative segments of the intracranial, lateral ventricles, choroid plexus, posterior cranial fossa, cerebellum, and brainstem, obtained from the presented CT images, are displayed in different colors in the lower row (**b**)

In this study, the ROI focused on intracranial volume. Representative intracranial segmentations from a single subject, along with the original CT images, are shown in Fig. 2. If desired, our method can also be applied to segment ROIs beyond the intracranial volume, such as the lateral ventricles, choroid plexus, posterior cranial fossa, cerebellum, and brainstem (Fig. 3). Results obtained using our method for the lateral ventricles and choroid plexus have been published previously [6, 18], as have results for the posterior cranial fossa, cerebellum, and brainstem [5, 9].

### Volume calculation using osirix

The.dcm files generated by the method described above were imported into OsiriX. The OsiriX display was mirrored onto a tablet computer, where the ROI was manually traced using a stylus pen to obtain segmentation, following the same methodology described above. A single examiner (Hiroaki Hashimoto) also performed the segmentation process. After ROI segmentation, OsiriX calculates the ROI volume (mL).

### Statistical analyses

**Fig. 4:**
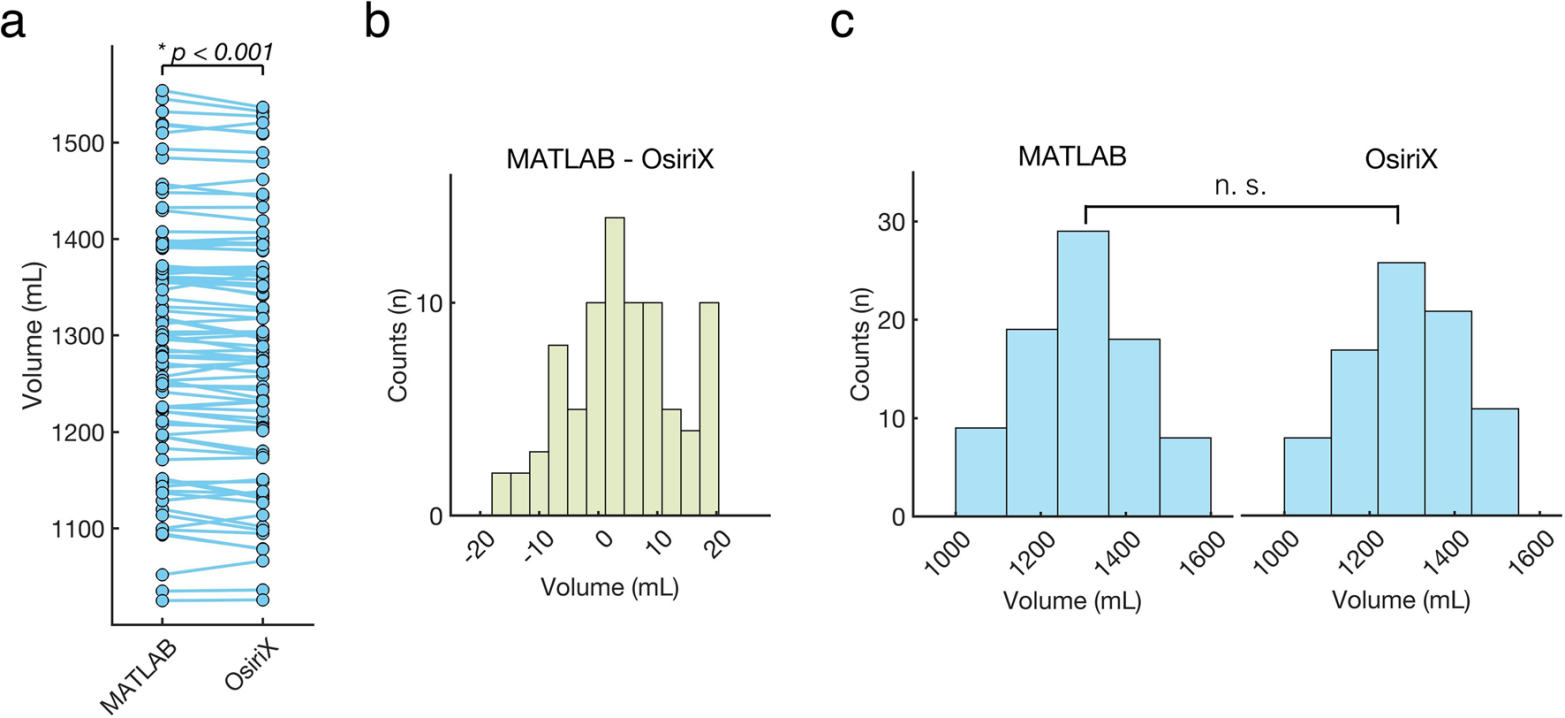
Results of statistical analyses. (**a**) Volumetric values calculated with our MATLAB method and OsiriX for each patient are connected by solid lines. A paired t-test reveals a statistically significant difference between the MATLAB method and OsiriX (*p* < 0.001). (**b**) Differences between methods were calculated by subtracting OsiriX values from MATLAB values for each patient (MATLAB—OsiriX), with the distribution of these differences shown. (**c**) Histograms depict the distributions of volumetric values obtained with our MATLAB method and OsiriX. An unpaired t-test, assuming the groups are independent, shows no significant (n.s.) difference between the two groups

We generated two sets of volumetric data using our MATLAB-based method and Osirix, both derived from the same subject population and CT scans. For each subject, two different volumetric values were calculated, one from the MATLAB method and the other from OsiriX. Since these two data sets correspond to the same subjects, a paired t-test was performed to assess the differences between the two methods. This test evaluates whether the volumetric measurements obtained from the MATLAB method significantly differ from those obtained with OsiriX Fig. 4.

In addition, an unpaired t-test was conducted to explore potential differences in volumetric datasets, treating them as independent samples from the same underlying population obtained using two distinct methodologies. This approach tested whether the MATLAB method and OsiriX produced significantly different volumetric data sets.

Statistical significance was defined as *p*-values < 0.05. Additionally, a power analysis was conducted to determine the effect size (Cohen’s d) and power (1—beta).

## Results

### Baseline characteristics

A total of 83 CT scans from pediatric patients with minor head trauma were analyzed according to the exclusion and inclusion criteria, with 33 females (39.8%). The mean age at the time of the CT scans was 66.55 ± 30.72 months. The volumetric values calculated using our MATLAB method had a mean of 1292.68 ± 128.02mL. The volumetric values calculated using OsiriX had a mean of 1288.60 ± 128.03mL.

### Results from paired T-test

We compared the volumetric values calculated using our MATLAB method with those from OsiriX using a paired t-test. A significant difference was observed (*p* < 0.001, Fig. 4a), indicating that the volumetric values obtained using our MATLAB method were statistically different from those obtained using OsiriX. The difference between values obtained from our MATLAB method and those from OsiriX for each patient had a mean of 4.09 ± 9.12 mL. The distribution of these differences is shown in Fig. 4b. On average, the volumetric values from our MATLAB method were 4.09 mL higher than those from OsiriX. This corresponds to 0.32% of the intracranial volume.

### Results from unpaired T-test

The distributions of volumetric values from our MATLAB method and OsiriX are presented in Fig. 4c. An unpaired t-test revealed no statistically significant difference between the two groups (p = 0.84). This suggests that, when treated as independent samples, the volumetric groups produced by our MATLAB method and Osirix did not differ significantly.

### Power analysis

The results of the power analysis for the paired and unpaired t-tests used in this study are shown in Table 1. The power of the paired t-test was high (0.98), confirming that the volumetric values obtained using our MATLAB method were statistically different from those obtained using OsiriX. However, the power of the unpaired t-test was low (0.06), due to the small effect size (0.032). The sample size required for a power of 0.8 was estimated to be 7709. The beta error was calculated as 0.94 (1 – 0.06), indicating that there is a high risk of incorrectly accepting the null hypothesis in the unpaired t-test.

**Table 1:** Results of power analysis

Test Type	Effect size (Cohen’s d)	Sample size for 0.8 power	Power (1—beta)
Paired t-test	0.44	42	0.98
Unpaired t-test	0.032	7709	0.060

For both paired and unpaired t-test conditions, the effect size was calculated based on Cohen’s d, the required sample size to achieve a power of 0.8 was determined, and the power values were calculated

## Discussion

This study introduces a methodology for calculating intracranial volume from head CT DICOM data using custom MATLAB code, a tablet computer, and a stylus pen. To evaluate the accuracy of our MATLAB-based method, we compared it with an established method, OsiriX. The paired t-test revealed a significant difference between the two methods, with the volumetric values obtained using our MATLAB method being 0.32% larger than those from OsiriX.

OsiriX is a well-established DICOM viewer [19], recognized for its user-friendly interface, efficient image loading capabilities, and its availability as open-source and free software [20]. Given its widespread use in volumetric assessments [21–23], OsiriX was selected as the reference for evaluating the accuracy of our MATLAB-based method. While we initially anticipated no significant differences between the results from our method and OsiriX, the paired t-test revealed a statistically significant discrepancy.

One potential explanation for this difference is the manual segmentation of ROIs, which may have introduced slight variations between the areas segmented using our method and OsiriX; however, since all segmentations were performed by a single examiner (Hiroaki Hashimoto), the variation due to manual segmentation was consistent across all samples, and we believe that the effects of this variability were minimized by analyzing a larger sample size. Therefore, we infer that the observed statistical difference is more likely attributable to methodological differences rather than segmentation-related variation. While we cannot definitively explain the cause of this difference, we noticed that manually segmented ROIs tended to appear slightly smaller in OsiriX compared to our MATLAB method. This suggests that the measurements obtained using Osirix were, on average, 4.09 mL smaller than those obtained using our method. However, this remains an assumption.

Conversely, the unpaired t-test demonstrated no statistically significant differences between the two volumetric groups. While this does not imply that the groups are identical, it suggests that no meaningful statistical differences exist between the volumetric groups obtained by the two methods. This finding further indicates that both volumetric populations likely originate from the same underlying population. However, attention must be paid to its low statistical power, such as 0.060. Based on these results and considerations, our MATLAB-based method has the potential to serve as a valuable and clinically useful tool.

In our previous research, we used this MATLAB-based method to calculate volumes of various structures. For example, using head MRI, we quantified lesion volumes on diffusion-weighted imaging, demonstrating differential outcomes between groups undergoing mechanical thrombectomy [24]. Similarly, using head CT, we measured chronic subdural hematoma volumes, identifying differences between recurrence and non-recurrence groups [25]. Additionally, we have published several studies investigating intracranial structural volumes in the Japanese pediatric population [5, 6, 9, 18]. In this study, while we focused on intracranial volume using head CT images, our method can also be applied to MRI DICOM data. Moreover, manual segmentation enables the measurement of regions beyond the intracranial volume. We suggest that readers consider using this method to segment various normal structures as well as pathological lesions, such as tumors, from CT or MRI DICOM data.

Manual segmentation, as employed in our MATLAB method, requires additional time and effort. To mitigate this issue, we proposed using a stylus pen for tracing segmented lines on a tablet computer screen, which mirrors the desktop display. This approach has been reported previously [26], showing that stylus pen-based volumetric techniques offer a technological advancement over traditional mouse-based tracing by providing improved statistical accuracy and significantly reducing the time required [27]. Therefore, we recommend using our MATLAB method in combination with a tablet computer and a stylus pen to enhance efficiency and accuracy.

This study used a 5 mm slice width for volume calculation. However, CT images with a 1 mm slice width are now widely used at our hospital, allowing for more accurate image evaluation. At our institution, head CT images are initially acquired with a 0.5 mm slice width and then reconstructed into 5 mm or 1 mm slices. While 5 mm slices are routinely reconstructed in all cases, 1mm slices are only reconstructed when necessary to avoid unnecessary procedures. Additionally, as 1 mm slice images contain five times more data than 5 mm slices, the labor and time required for manual segmentation would be significantly increased. Since the primary goal of this study was to compare our MATLAB method with OsiriX, we opted to use 5mm slice CT images to minimize the time and effort needed for segmentation.

This study has several limitations. The primary limitation is that a single examiner segmented all samples. This approach was chosen to maintain consistency in manual segmentation and minimize variability. However, to develop a robust volumetric calculation utility, multiple examiners should evaluate and validate the method. For instance, interobserver variability could be assessed using Cohen’s kappa [28]. Without such assessments, the wider applicability of the method cannot be established. This study focused on comparing our method with an established method, such as OsiriX. We thus concluded that segmentation by a single examiner was acceptable for this specific evaluation. Second, this study exclusively focused on volumetric measurements derived from segmentation. Consequently, we did not evaluate Hounsfiled units within the segmented regions, which are commonly assessed using voxel-wise analysis [29]. While voxel-wise analysis could benefit the development of the methodology, further studies are needed to explore its application. Additionally, due to the retrospective nature of this study, different CT protocols were used, and we could not ensure uniformity across these protocols. Furthermore, while 5-mm slice thickness CT images were used, more accurate volumetric evaluations could likely be achieved with 1-mm slice images.

## Conclusions

This study presented the MATLAB-based methodology for calculating intracranial volume using a tablet computer and stylus pen, which was compared with OsiriX. The results of the paired t-test, showing significant differences, likely reflect discrepancies in calculation methodologies. Conversely, the results of the unpaired t-test, which showed no significant differences, indicate that the volumetric populations derived from our method and OsiriX may originate from the same underlying population. The MATLAB code used in this study is provided in the Supplemental Data, enabling readers to implement our method easily.

## Supplementary Information

Below is the link to the electronic supplementary material.

Supplementary file1 (DOCX 27 KB)

## Data Availability

No datasets were generated or analysed during the current study.

## Abbreviations

CD-R: Compact disc-recordable
CT: Computed tomography
DICOM: Digital Imaging and Communications in Medicine
MATLAB: MATrix LABoratory
mL: Milliliter
MRI: Magnetic resonance imaging
ROI: Regions of interest
