# Supplementary material for: Methodology for pediatric head computed tomography image segmentation and volumetric calculation using a tablet computer and stylus pen
Source: Childs Nerv Syst. 2024 Dec 23;41(1):66. doi: 10.1007/s00381-024-06723-y (PMC11666657; doi:10.1007/s00381-024-06723-y)
Supplement: Supplementary file 1 — Supplementary file1 (DOCX 27 KB) [file 381_2024_6723_MOESM1_ESM.docx]

**Supplemental Table 1.** CT scan protocols utilized in this study.

The CT scans evaluated in this study were acquired using several distinct protocols, as outlined below. Due to the lack of available records before 2012, data from five scans are missing. For pediatric patients with difficulty controlling body movement, a volume scan protocol was used. Helical scans are categorized by tube current, rotation time, pitch factor, and whether auto exposure control (AEC) was employed. Radiation doses are reported as the mean volume computed tomography dose index (CTDI_vol_), with standard deviation. All CT scans were acquired with a 0.5mm slice width, and images were reconstructed with a 5 mm slice thickness.

AP, Aquilion Prime SP; AO, Aquilion ONE.

| **Scanning Type** | **Scanner Model** | **Tube Voltage [kV]** | **Tube Current**  **[mA]** | **Rotation Time**  **[s]** | **Pitch Factor**  **[mm]** | **Collimation**  **[mm]** | **Slice Thickness**  **[mm]** | **AEC** | **CTDI_vol_**  **[mGy]** |
| --- | --- | --- | --- | --- | --- | --- | --- | --- | --- |
| **Helical**  **(n = 1)** | AP | 120 | 150 | 0.6 | 0.638 | 0.5 × 64 | 5 | On | 17.50 ± 0.00 |
| **Helical**  **(n = 15)** | AO | 120 | 150 | 0.6 | 0.641 | 0.5 × 64 | 5 | On | 21.01 ± 1.08 |
| **Helical**  **(n = 1)** | AO | 120 | 170 | 0.6 | 0.641 | 0.5 × 64 | 5 | On | 23.10 ± 0.00 |
| **Helical**  **(n = 15)** | AP | 120 | 200 | 0.6 | 0.638 | 0.5 × 64 | 5 | On | 19.09 ± 1.62 |
| **Helical**  **(n = 5)** | AO | 120 | 200 | 0.6 | 0.641 | 0.5 × 64 | 5 | Off | 37.40 ± 1.82 |
| **Helical**  **(n = 34)** | AO | 120 | 200 | 0.6 | 0.641 | 0.5 × 64 | 5 | On | 29.29 ± 2.65 |
| **Helical**  **(n = 1)** | AO | 120 | 220 | 0.75 | 0.641 | 0.5 × 64 | 5 | On | 34.80 ± 0.00 |
| **Helical**  **(n = 1)** | AO | 120 | 230 | 0.6 | 0.641 | 0.5 × 64 | 5 | On | 28.60 ± 0.00 |
| **Helical**  **(n = 1)** | AP | 120 | 230 | 0.75 | 0.638 | 0.5 × 64 | 5 | On | 20.50 ± 0.00 |
| **Helical**  **(n = 1)** | AP | 120 | 300 | 0.6 | 0.638 | 0.5 × 64 | 5 | On | 16.60 ± 0.00 |
| **Helical**  **(n = 1)** | AP | 120 | 300 | 0.75 | 0.638 | 0.5 × 64 | 5 | On | 31.50 ± 0.00 |
| **Helical**  **(n = 1)** | AO | 120 | 300 | 0.75 | 0.641 | 0.5 × 64 | 5 | On | 55.10 ± 0.00 |
| **Volume**  **(n = 1)** | AO | 120 | 100 | 1 | 1 | 0.5 × 320 | 5 | Off | 19.90 ± 0.00 |

**Supplementary code**

We have provided the MATLAB code used in the study. This code enables you to segment regions of interest (ROI) in digital imaging and communications in medicine (DICOM) images and calculate the volume of the ROI. Please copy and paste the code below into the MATLAB editor.

%% Supplementary code written by Hiroaki Hashimoto on 03/21/2024.

% If you have multiple DICOM images from a single CT or MRI examination for one subject,

% please save them as a single file (.dcm) using Mango software.

% https://mangoviewer.com

% Launch Mango, and navigate to the folder where the DICOM files are saved.

% Export the DICOM data as .dcm files.

%load data

[a,b] = uigetfile('.dcm');

cd(b);

DICOMd = dicomread(a);

DICOMd = squeeze(DICOMd);

DICOMinf = dicominfo(a);

fprintf([a,' was loaded. \n'])

Tn = size(DICOMd,3);

% Index for displaying DICOM images.

% Please adjust them to display them appropriately

RangeMin = 32770;

RangeMax = 32850;

F1 = figure;

F1.Position = [1 1061 1693 726];

subplot(1,2,1);

montage(DICOMd, 'DisplayRange', [RangeMin RangeMax])

title('Original Image')

DICOMd2 = nan(size(DICOMd));

for i = 1:Tn

CurrD = DICOMd(:,:,i);

CurrD(CurrD<RangeMin) = RangeMin;

CurrD(CurrD>RangeMax) = RangeMax;

DICOMd2(:,:, i) = CurrD;

end

subplot(1,2,2);

montage(DICOMd2, 'DisplayRange', [RangeMin RangeMax])

title('Modified Image')

%% Segmentation

fprintf('Please export segmentations as BW1, BW2, BW3, etc., to the workspace.\n')

fprintf('Caution: The numbering of BW variables must match the number of slices.\n\n')

fprintf('For example, when segmenting DICOM slice No.7, the corresponding BW must be renamed as BW7.\n\n')

fprintf('When there are no ROIs or you have finished exporting the ROIs to the workspace, \nplease close the window and press Enter in the command window.\n\n')

for i =1:Tn

fprintf('DICOM slice No.%d is displayed. (Rest: %d)\n',i, Tn-i)

fprintf('If there are any ROIs, please manually segment them and export as BW%d.\n', i)

imageSegmenter(DICOMd2(:,:,i))

pause

end

%% Combine separate BW variables into one variable.

VarList = who;

Varnum = size(VarList,1);

NoDataMat = zeros(size(DICOMd,1), size(DICOMd,2));

SegmentationMat = nan(size(DICOMd));

for i = 1:Tn

eval(['SelectNum = {''BW', num2str(i), '''};'])

YN = 0;

for l = 1:Varnum

CompVar = VarList{l,1};

if strcmp(SelectNum, CompVar)

YN = YN +1;

end

end

if YN == 0

eval(['BW', num2str(i), ' = NoDataMat;'])

end

eval(['SegmentationMat(:,:,i) = BW', num2str(i), ';'])

end

F2 = figure;

F2.Position =[1 256 1694 725];

subplot(1,2,1);

montage(DICOMd2, 'DisplayRange', [RangeMin RangeMax])

title('Modified Image')

subplot(1,2,2);

montage(SegmentationMat, 'DisplayRange', [0 1])

title('Segmentation Image')

%Calculation of volume.

volD = regionprops3(SegmentationMat,'volume');

SegmentVol = volD{:,:} * DICOMinf.SliceThickness * DICOMinf.PixelSpacing(1,1) * DICOMinf.PixelSpacing(2,1) * 1e-3 ;

fprintf('The volume of segmentation is %.2fmL.\n', SegmentVol)
